# The typification of *Cordia flavescens* Aubl., the transfer of *Firensia* Scop. from *Cordia* L. (Cordiaceae, Boraginales) to the synonymy of *Ocotea* Aubl. (Lauraceae), and the identity of the species of *Firensia*

**DOI:** 10.3897/phytokeys.23.4827

**Published:** 2013-05-17

**Authors:** Christian Feuillet

**Affiliations:** 1 Department of Botany, MRC–166, National Museum of Natural History, Smithsonian Institution, P.O. Box 37012, Washington, DC 20013-7012, USA

**Keywords:** Aublet, Boraginales, Combretaceae, *Cordia*, Lauraceae, French Guiana, Rafinesque, typification

## Abstract

*Firensia* Scop. was based on *Cordia flavescens* Aubl., a species described and illustrated from a mixed collection that Scopoli never transferred to *Firensia*. The genus included three additional species formally named by Rafinesque. Currently the four species are placed in three different families and none retained the epithet accepted by Scopoli or given by Rafinesque for reason of priority. A lectotype is designated for *Cordia flavescens* that places *Firensia* in the synonymy of *Ocotea* (Lauraceae).

## Introduction

In the preparation of the treatment of the Boraginaceae for the Flora of the Guianas, I got intrigued by the history of *Firensia* Scop. When Scopoli (1777: 157) described *Firensia*, he included only *Cordia flavescens* Aubl. that is therefore the type of the genus. Contrary to what is stated by Jackson (Index Kewensis 1893, as reflected in IPNI (http://www.ipni.org/index.html; accessed 23.01.2013), in that work *Cordia flavescens* was not transferred to *Firensia*. Necker (1790, 1: 276) accepted *Firensia* as one of his “*species naturalis*” in a work where the nomenclature was uninominal and that is rejected as a source of names by the International Code of Botanical Nomenclature (McNeill et al. 2006, App. VI: 483). Three species were placed in *Firensia* by Rafinesque (1838: 40), two of them illegitimate renaming. Other authors ignored *Firensia* or placed it in the synonymy of *Cordia* (ex.: Candolle 1845: 471 [as “Firenzia”]). Then Kuntze (1891, 2: 977) placed them in *Lithocardium* “L. 1735”. As this is a pre-1753 name, he created *Lithocardium* Kuntze with *Cordia* L. as a synonym. It is an illegitimate renaming of *Cordia*.

## Methods

To solve the question of the generic and familial placement of Firensia, I studied the literature and type specimens. I looked also at printed photographs found in the cited herbarium collections, and examined scanned of specimens or photos of specimens posted online, available directly through the herbarium site, or through the sites of JSTOR or Europeana. Example: “photos F, MO!, US!; scan!”. The Internet address to the scans is given in a note below the species.

A few typifications had to be made. When the type collection was known, but not the holotype, the text says: “Type:... (lectotype... ; isotypes...)”. When the original description was associated with several collections of equal status or syntypes, a type collection and a lectotype were selected and the text says: “Lectotype...: ... (hololectotype...; isolectotypes...)”. In both cases the information on the date of lectotypification is given next to the word lectotype.

## Taxonomic treatment

### Typification of *Cordia flavescens* and familial placement of *Firensia* in the Lauraceae

The name *Cordia flavescens* Aubl. was described and illustrated based on a mixture of fruiting branches of *Ocotea commutata* Nees (LAURACEAE) and flowers belonging in *Cordia*. Specimens by Aublet at BM and S represent only the *Ocotea* element (Johnston 1935: 44). This offers an opportunity to give a clear identity to the name by designating as the lectotype a specimen lacking the *Cordia* element.

*Cordia flavescens* *Aubl*. is the only species included in *Firensia* by Scopoli (1777: 157) and is therefore the type species of *Firensia* Scop. *Firensia* falls in the synonymy of *Ocotea* Aubl. 1775 and belongs in the Lauraceae.

Nomenclature stability is satisfied: *Firensia* Scop. (1777) does not have priority over *Ocotea* Aubl. (1775), and the epithet *flavescens* cannot be transferred to *Ocotea* as the name *Ocotea flavescens* Rusby (1920) applies to a different species from Colombia. Aublet’s name becomes a synonym of *Ocotea commutata* (Nees) Mez.

***Ocotea* Aubl., Hist. Pl. Guiane 2: 780; 4: t. 310. 1775.**

Type: *Ocotea guianensis* Aubl.

*Firensia* Scop., Intr. Hist. Nat. 157. 1777.

Type: *Cordia flavescens* Aubl.

### Identity of the type species and those once placed in *Firensia*

The species once placed in *Firensia* have been named or identified as species of *Cordia* L., one by Linnaeus (1757: 14) and three by Aublet (1775: 219, 224, 226; t. 86, 88, 89). None of the epithets used in *Firensia* could be kept for reason of priority. The type species, *Cordia flavescens*, and the three species of *Firensia* currently belong in three different genera and families.

#### 1. Cordia flavescens

Aubl., Lauraceae

Ocotea commutata (Nees) Mez, Jahrb. Königl. Bot. Gart. Berlin 5: 327. 1889. Type: Based on *Oreodaphne commutata* Nees
Cordia flavescens Aubl., Hist. Pl. Guiane 1: 226; 3: t. 89. 1775; not *Ocotea flavescens* Rusby 1920. Type: French Guiana. *J.B. Aublet s.n*. [specimens without the *Cordia* flowers](lectotype here designated BM [BM-000993950; scan!]; isotypes LINN [SM 374.6], S [04-2350; scan!])
Cordia sarmentosa Lam., Tab. Encycl. Méthod. 1: 422. 1791; illegitimate renaming. Type: Based on *Cordia flavescens* Aubl.
Oreodaphne commutata Nees, Syst. Laur. 428. 1836. Type: French Guiana. *J. Martin s.n*. (holotype B “ex P” [photos neg. F-3643, MO!, US!; scan!]; isotypes B [2 sheets, scans!], K, P! [2 sheets, scans!] and as “*Martin 20*” P! [scan!]
Lithocardium flavescens (Aubl.) Kuntze, Revis. Gen. Pl. 2: 977. 1891. Type: Based on *Cordia flavescens* Aubl.
Gerascanthus flavescens (Aubl.) Borhidi, Acta Bot. Hung. 34(3–4): 400. 1988. Type: Based on *Cordia flavescens* Aubl.

##### Note.

The holotype of *Oreodaphne commutata* can be seen at http://ww2.bgbm.org/herbarium/view_large.cfm?SpecimenPK=47688&idThumb=253360&SpecimenSequenz=1&loan=0 , and the Paris isotypes at http://coldb.mnhn.fr/colweb/request.do?requestaction=exec; the lectotype of *Cordia flavescens* at http://plants.jstor.org/specimen/bm000993950 , and its Stockholm isotype at http://plants.jstor.org/specimen/s04-2350?history=true .

#### 2. Firensia fusca

Raf., Cordiaceae

Cordia collococca L., Sp. Pl. ed. 2, 1: 274. 1762. Lectotype (designated by Miller 1988, 1999): Jamaica. *P. Browne s.n*. (hololectotype LINN, Savage Catalog number 253.8[scan!])
Firensia fusca Raf., Sylva Tellur. 40. 1838; illegitimate renaming. Type: Based on *Cordia collococca* L.
Lithocardium collococca (L.) Kuntze, Revis. Gen. Pl. 2: 438. 1891. Type: Based on *Cordia collococca* L.
Gerascanthus collococcus (L.) Borhidi, Acta Bot. Hung. 34(3–4): 399. 1988. Type: Based on *Cordia collococca* L.

##### Notes.

The hololectotype of *Cordia collococca* can be seen at [herb. LINN scan!; http://www.linnean-online.org/view/plants_alpha/cordia_callococca.html; accessed 22.01.2013].

For other synonyms, see Feuillet (2012: 160).

#### 3. Firensia hirsuta

(Willd.) Raf., Cordiaceae

Cordia nodosa Lam., Tab. Encycl. 1: 422. 1792. Type: Aublet (1775) Hist. Pl. Guiane 3: pl. 86.
Cordia collococca sensu J.B. Aublet, Hist. Pl. Guiane 1: 219; & 3: pl. 86. 1775; non L. 1757. (= *Cordia nodosa* Lam.)
Cordia hirsuta Willd., Sp. Pl., ed. 5 [as “4”] 1(2): 1076. 1798. Type: Based on *Cordia nodosa* Lam.
Firensia hirsuta (Willd.) Raf., Sylva Tellur. 40. 1838. Type: Based on *Cordia nodosa* Lam.
Lithocardium nodosum (Lam.) Kuntze, Rev. Gen. 2: 977. 1891. Type: Based on *Cordia nodosa* Lam.

##### Notes.

Sometimes the collection from French Guiana—J.B. Aublet s.n. (BM [scan!], LINN SM-374.5 [microfiche!], P-Rousseau 5: 181! [acquired by P only in 1953])—is cited as the type of *Cordia nodosa* and *Cordia hirsuta*. In fact it is unlikely that Lamarck (1792, 1, 2(1): 422) or Willdenow (1798, 1(2): 1076) saw a specimen as they only cited Aublet plate 86.

Aublet s.n. can be seen at http://plants.jstor.org/specimen/bm000906214

*Cordia hirsuta* Fresen. 1857 is different from *Cordia hirsuta* Willd. 1798.

For other synonyms, see Johnston (1935: 13–14).

#### 4. Firensia lutea

Raf., Combretaceae

Buchenavia tetraphylla (Aubl.) R.A. Howard, J. Arnold Arbor. 64(2): 266. 1983. Type: Based on *Cordia tetraphylla* Aubl.
Cordia tetraphylla Aubl., Hist. Pl. Guiane 1: 224; 3: t. 88. 1775. Lectotype (designated by Howard 1983: 266): Plate, Aubl., Hist. Pl. Guiane 1: pl. 88, excl. f. 1-3 (1775).
Firensia lutea Raf., Sylva Tellur. 40. 1838; illegitimate renaming. Type: Based on *Cordia tetraphylla* Aubl.
Lithocardium tetraphyllum (Aubl.) Kuntze, Rev. Gen. 2: 977. 1891. Type: Based on *Cordia tetraphylla* Aubl.
Gerascanthus tetraphyllus (Aubl.) Borhidi, Acta Bot. Hung. 34(3–4): 402. 1988. Type: Based on *Cordia tetraphylla* Aubl.

##### Note.

For other synonyms, see Acevedo and Strong (2012: 229).

### Invalid names

*Ocotea commutata* Nees ex Meisn., Prodr. (DC.) 15(1): 120. 1864, invalid: pro syn. *Oreodaphne commutata* Nees (= *Ocotea commutata* (Nees) Mez 1889).

*Cordia echitioides* Lam. ex D. Dietr., Syn. Pl. 1: 612. 1839; invalid: pro syn. *Cordia flavescens* Aubl. (= *Ocotea commutata* (Nees) Mez).

## Supplementary Material

XML Treatment for Cordia flavescens

XML Treatment for Firensia fusca

XML Treatment for Firensia hirsuta

XML Treatment for Firensia lutea

## References

[B1] Acevedo-Rodríguez P Strong MT (2012) Catalogue of Seed Plants of the West Indies. Smithsonian Institution Scholarly Press, Washington, 1–1192. http://hdl.handle.net/10088/17551

[B2] Aublet JB (1775) Histoire des Plantes de la Guiane Françoise. Didot jeune, London, Paris, 4 vol., 1–976 & plates 1–392. 10.5962/bhl.title.674

[B3] Candolle AP de (1845) Prodromus Systematis Naturalis Regni Vegetabilis, vol. 9. Treuttel et Wurtz, Paris. 1–573. 10.5962/bhl.title.286

[B4] Feuillet C (2012) Boraginaceae. In: Acevedo-Rodríguez P, Strong MT (Eds) Catalogue of Seed Plants of the West Indies. Smithsonian Institution Scholarly Press, Washington, 157–174. http://hdl.handle.net/10088/17551

[B5] Howard RA (1983) The plates of Aublet’s Histoire des Plantes de la Guiane Françoise. Journal of the Arnold Arboretum 64(2): 255–292. http://www.biodiversitylibrary.org/item/33627

[B6] Jackson BD (1893) Index Kewensis vol. 1. Oxford, 1–1268.

[B7] Johnston IM (1935) Studies in Boraginaceae X. The Boraginaceae of northeastern South America. Journal of the Arnold Arboretum 16(1): 1–64. http://www.biodiversitylibrary.org/item/33592

[B8] Kuntze O (1891) Revisio Generum Plantarum vol. 2. Arthur Felix, Leipzig, 377–1011. 10.5962/bhl.title.327

[B9] Lamarck JB (1792) Tableau Encyclopédique et Méthodique des Trois Règnes de la Nature, Botanique tome 1, vol. 2, part 1. Panckoucke, Paris, 353–440. 10.5962/bhl.title.824

[B10] Linnaeus C (1757) Flora Jamaicensis. Sandmark, Upsala, 1–27. 10.5962/bhl.title.4340

[B11] McNeill J Barrie FR Burdet HM Demoulin V Hawksworth DL Marhold K Nicolson DH Prado J Silva PC Skog JE Wiersema JH Turland NJ (eds) (2006) International Code of Botanical Nomenclature (Vienna Code). Regnum Veg. 146. Gantner Verlag, Ruggell, 1–568. http://ibot.sav.sk/icbn/main.htm

[B12] McNeill J Barrie FR Buck WR Demoulin V Greuter W Hawksworth DL Herendeen PS Knapp S Marhold K Prado J Prud’Homme van Reine WF Smith GF Wiersema JH Turland NJ (2012) International Code of Nomenclatureforalgae, fungi, and plants(Melbourne Code). http://www.iapt-taxon.org/nomen/main.php?page=title

[B13] Miller JS (1988) A revised treatment of Boraginaceae for Panama. Annals of the Missouri Botanical Garden 75: 456–521. http://www.biodiversitylibrary.org/item/87375 , 10.2307/2399433

[B14] Miller JS (1999) New Boraginaceae from Tropical America 1: New species of *Bourreria* and *Tournefortia* from Costa Rica and a note on the publication of *Cordia collococca*. Novon 9 (2): 230-235.10.5962/bhl.title.744

[B15] Necker NJ (1790) Firensia. Elementa Botanica 1: 276. 10.5962/bhl.title.4452

[B16] Rafinesque CS (1838) Sylva Telluriana, part 1. 184 pages. Philadelphia, 1–184. 10.5962/bhl.title.248

[B17] Scopoli JA (1777) Introductio ad Historiam Naturalem. Gerle, Prague, 1–540. 10.5962/bhl.title.10827

[B18] Willdenow CL (1798) Species Plantarum , ed. 4, 1(2). Nauk, Berlin, 1–1070. 10.5962/bhl.title.60064

